# Advances in Substance Abuse Prevention and Treatment Interventions Among Racial, Ethnic, and Sexual Minority Populations

**DOI:** 10.35946/arcr.v38.1.06

**Published:** 2016

**Authors:** Arthur W. Blume

**Affiliations:** Arthur W. Blume, Ph.D., is a professor in the Department of Psychology at Washington State University, Vancouver, Washington

**Keywords:** Alcohol use, abuse, and dependence, alcohol research, race, ethnicity, minorities, ethnic minorities, sexual minorities, prevention, intervention, treatment, point of sale intervention, family intervention, computer technology, cultural traditions, culturally grounded intervention

## Abstract

Substance abuse research among racial, ethnic, and sexual minority populations historically has lagged behind that conducted with majority samples. However, interesting and potentially important advances in prevention, brief interventions, and treatment have been made in the last few years, at least among some minority populations, such as American Indian youth. New prevention efforts have focused on point-of-sale interventions for alcohol, as well as on family-unit interventions designed with subpopulation cultural values in mind. In addition, previously established evidence-based and culturally relevant interventions are being combined with computer technology. Empirical data support using brief interventions with patients of color in medical settings, capitalizing on teachable and reachable moments during a physical trauma or other health crisis. Finally, use of empirically supported treatment may be helpful, with a caveat that these interventions must appropriately match cultural traditions and respect the values of the clients. More research clearly is needed, especially among certain minority populations in the United States. A greater emphasis should be placed on developing novel, culturally grounded interventions in partnership with communities, in addition to adapting existing mainstream interventions for use by other cultures.

Historically, prevention and treatment intervention research rarely has been conducted with racial and ethnic or sexual minorities as its principal focus; this also holds true for the alcohol and other drug abuse field. The lack of credible research has been one source of the disparities in substance abuse and its consequences found among many of these groups. Fortunately, advances recently have been made in preventing, intervening in, and treating substance abuse among traditionally underserved racial, ethnic, and sexual minority subpopulations. This article reviews some of these advances, focusing on alcohol abuse but also including abuse of other drugs or substance abuse in general, as appropriate. The article also will suggest next steps for research in this area.

## Challenges in Addressing Prevention and Treatment for Minority Populations

Many minority populations in the United States face well-documented challenges, such as higher-than-average rates of poverty, homelessness, and incarceration, which may contribute to increased rates of alcohol use disorder as well as other substance use disorders. A less concrete factor influencing prevention and treatment is that minorities often face stereotypes in the general population. Such stereotypes foster biased behavior toward minority groups, which may promote alcohol and other drug abuse and create greater levels of anxiety among group members themselves (Blume et al. 2012). Such factors also are likely to affect whether members of minority groups decide to seek treatment and how they experience treatment if they do (for a review of access to treatment studies, see Schmidt in this issue).

Cultural background also figures into how minority populations respond to treatment and prevention efforts. Differences in worldviews, cultural traditions, and upbringing mean that not all groups may respond to an intervention that has demonstrated success in the general population (Taylor 2003). Certain groups also face specific challenges. For treatment to be effective, providers need to identify those challenges and offer appropriate interventions. For example, American Indian (AI) and Alaska Native (AN) populations face high rates of alcohol abuse among youth (SAMHSA 2014), and relatively easy access to alcohol may be one of the contributing factors. Thus, in one study (Lynne-Landsman et al. 2015) about 75 percent of all outlets tested sold alcohol to young-appearing AI buyers at least once. Other research confirmed that underage AI youth may obtain alcoholic beverages from stores both on and near reservations either directly through illegal sales to minors or indirectly through purchases by adult friends (Lee et al. 2015). Prevention efforts aimed at lowering sales of alcohol to minors therefore could be effective for these groups. For example, Moore and colleagues (2013) demonstrated that a reward-and-reminder underage drinking prevention program in convenience stores could reduce alcohol sales to minors near rural reservations.

Recent research focused on prevention and treatment efforts for minorities has suggested that feeling safe in the environment both inside and outside of treatment centers plays a pivotal role in the success of interventions. As is discussed below, when a group’s basic needs are met, group members are more likely to cut back on drinking (Larimer et al. 2009). Furthermore, when they feel secure—that is, understood culturally and not threatened—they express deeper satisfaction with treatment or prevention programs and may be more likely to continue participating (Guerrero 2013). In some cases, adapting empirically proven treatment methods is sufficient in helping clients feel safe; but in others, novel, culturally centered approaches may prove useful.

## Advances in Understanding the Treatment Environment

Various studies have highlighted the importance of a safe environment for positive treatment outcomes among clients from racial, ethnic, and sexual minority groups. The groundbreaking Housing First study demonstrated that a safe housing environment alone was sufficient to improve substance-use outcomes and reduce public health costs in people with severe alcohol problems, including many homeless people of color (Larimer et al. 2009). A more recent data analysis found that motivation to change predicted improved alcohol-use outcomes 2 years after the Housing First intervention, whereas attending abstinence-based treatment did not (Collins et al. 2012).

The prevention and treatment environment also affect substance abuse treatment outcomes through the therapeutic working alliance—that is, the working relationship that clients believe they have with their therapists. Positive working alliances have been found to predict successful treatment engagement and completion (Meier et al. 2005). Davis and Ancis (2012) pointed out that most studies investigating the working alliance in treatment have been conducted with predominately White patient samples. However, they did identify three important factors that affect the working alliance among clients of color. First, culturally responsive treatment has been positively associated with improvements in the working alliance. Second, in their interactions with both counselors and other treatment staff, clients of color encounter biased beliefs and attitudes, which often are the result of stereotyping. Third, poor working alliances frequently are a function of how often a client in therapy experiences microaggressions—commonly experienced insults, put-downs, or messages of exclusion stemming from stereotypes associated with minority-group membership—and of a client’s perceptions of a therapist’s low cultural competence.

**Figure 1:**
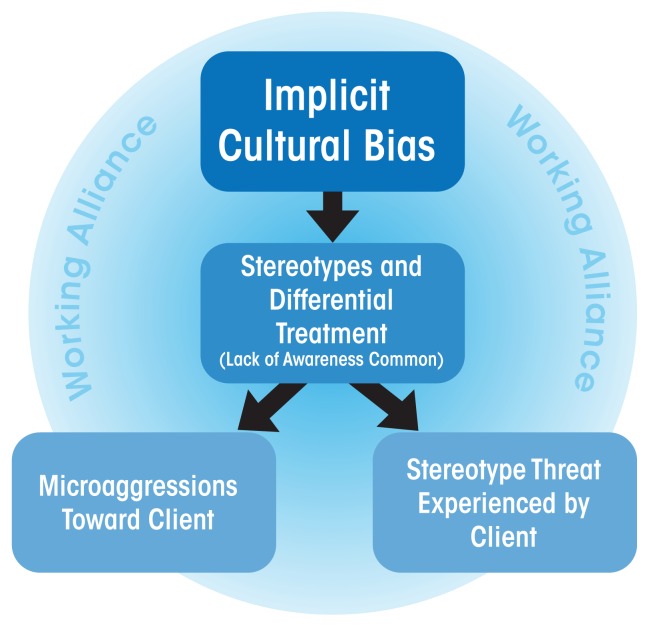
Implicit bias and its threat to working alliance. All people, including treatment professionals, are affected by implicit biases transmitted within our culture that may escape our personal awareness. Implicit bias makes the commission of microaggressions by staff and the experience of stereotype threat by minority clients more likely. This potentially harms the working alliance and undermines treatment outcomes.

Microaggressions correlate with alcohol abuse and greater anxiety (Blume et al. 2012). Thus, they may foster an environment conducive to alcohol problems and also may undermine the treatment environment and the working alliance. Microaggressions occur in the context of culturally implicit bias—that is, cultural biases ingrained in the social order that perpetuate stereotypes and prejudices often expressed automatically and without awareness by members of the social order (figure 1). Mental health professionals may direct microaggressions toward their clients automatically and unwittingly. Microaggressions also may result from programmatic or institutional cultural insensitivity toward clients (Sue et al. 2007). Interestingly, clients of color interpret the common lack of discussion in treatment concerning bias and prejudice and their links to substance-use behavior as a microaggression (Burris 2012).

Stereotyping also may influence substance-use and treatment outcomes by increasing the risk of stereotype-threat situations, in which minority members find themselves at risk for fulfilling a commonly held group-based stereotype (e.g., African Americans in academic situations where they are expected to perform poorly) (Steele and Aronson 1995). These situations place significant stress on minority-group members that can affect both physiological responses (e.g., blood pressure) (Blascovich et al. 2001) and cognitive function, including in substance abusers (Cole et al. 2006; Looby and Earleywine 2010). As an example, AI/AN clients often are stereotyped by the firewater myth, a belief that Native Americans cannot tolerate or regulate the ingestion of alcohol and will lose behavioral control if they drink any alcohol. AI/AN clients could experience stereotype-threat situations that may adversely affect treatment outcomes when treatment programs or professionals (perhaps unwittingly) communicate an understanding of addiction that aligns with the assumptions of the firewater myth.

The therapist is only one source of stereotyping and microaggression. The working alliance transcends the client–therapist relationship and includes the positive or negative impacts of institutional climate on clients. Indeed, discussions concerning prejudice and homophobia and their links to substance abuse have largely been ignored until very recently.

Research also has demonstrated that the cultural climate of treatment is a critical factor influencing treatment outcomes. Thus, increased cultural competence among treatment-center staff has been shown to contribute to higher rates of treatment retention (Guerrero 2013). Similarly, improved cultural sensitivity among treatment-program managers has been positively associated with higher rates of retention and less time on waitlists before treatment admission (Guerrero and Andrews 2011). Increasing the cultural competence of treatment administrators, counselors, and treatment-center staff who interact with clients seems to be one method for improving treatment outcomes, perhaps by making it less likely that clients will experience microaggressions and stereotype-threat situations.

## Matching and Molding Prevention and Treatment Interventions

In addition to evaluating the impact of the treatment environment, investigators have focused on determining which alcohol-related interventions facilitate success for minority clients. Recent studies in both prevention and treatment show that some mainstream interventions may be effective when matched with certain population subgroups in culturally appropriate ways. Moreover, their success often improves when adapted for use in different cultures.

Moving beyond such adaptations, some research suggests that creating new prevention and treatment methods with the participation of minority-group members can foster the success of interventions even more (Bermúdez Parsai et al. 2011; De las Nueces et al. 2012; Stacciarini et al. 2011; Tapp et al. 2013). Community-based participatory research (CBPR) methods, a research model that respects minority-community authority, needs, and values in the conduct of research, makes community stakeholders equal partners with scientists during all phases of project development, implementation, and dissemination. CBPR can be used to create novel interventions specifically tailored for racial and ethnic minority communities. The following sections focusing on prevention and treatment studies, respectively, demonstrate that all three approaches— matching existing methods in culturally relevant ways to the values and needs of the communities being served, adapting existing methods to different cultures, and creating new strategies with the participation of the target community—are demonstrating success in addressing alcohol problems among minority clients.

### Advances in Prevention

Over the last few years, researchers have begun developing and sometimes adapting prevention programs aimed at addressing problems specific to target populations and testing the programs empirically. One promising intervention targeted the availability of alcohol to underage purchasers near AI reservations in California. The reward-and-reminder program enlisted young-looking confederates who attempted to purchase alcohol without showing proper identification. When convenience-store clerks requested identification, they were rewarded with gift cards; when they did not, they were sent reminder letters concerning State laws about liquor sales. After two cycles of rewards and reminders, stores were completely in compliance when assessed (Moore et al. 2012).

**Figure 2:**
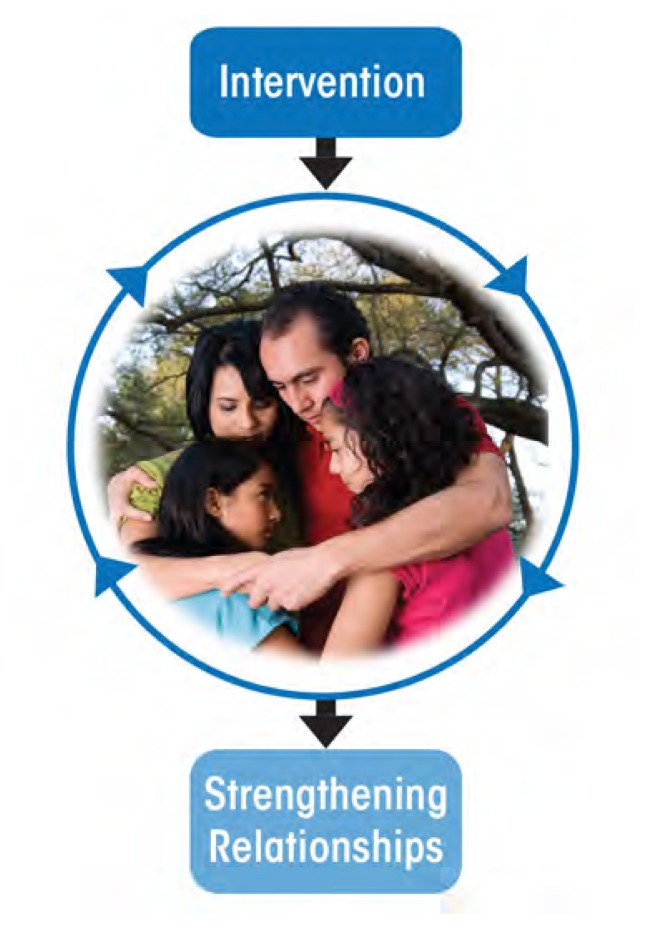
Family-oriented interventions. Recent advances in effective prevention programs among subpopulations have focused on family-level interventions consistent with the strong cultural values about the importance of family in collectively addressing the needs of a family member.

Culturally relevant prevention programs that focus on the family rather than on individuals have been successful, because they acknowledge beliefs held by many minority cultures concerning the importance of the family (rather than the individual) as the principal unit of function (figure 2). This family-oriented approach stresses the value of interdependence and the commonly held tenet that families work together to solve the problems of individual members. These interventions generally involve family members and parent–youth dyads working in unison on various family-building strategies (e.g., family communication) and substance-use prevention program components (e.g., parental monitoring). Other approaches include completing the more traditional individualized prevention components, such as parent training (for adults) or drink-refusal skills (for youth).

One family-oriented intervention, for example, targeted mother–daughter dyads through a Web-based delivery system. The investigators found reduced substance use, improved child–parent relationships, and increased self-efficacy and refusal skills among female adolescent African Americans, Asian Americans, and Latinas (Fang et al. 2010; Schinke et al. 2011). Other examples include the Familias Unidas program with Latino youth in the juvenile justice system and their primary caretakers, which led to a drop in substance abuse as well as in high-risk sex (Prado et al. 2012). The Strong African American Families and Adults in the Making programs resulted in slower increases in alcohol consumption and intoxication (i.e., slower alcohol-use escalation) among African-American youth compared with control subjects (Brody et al. 2010, 2012).

Skill-based interventions that incorporate traditional practices to strengthen the bonds of youth to their communities and cultures also are under investigation. Komro and colleagues (2015) are conducting a promising screening, brief intervention, and referral to treatment (SBIRT) prevention trial that includes a culturally centered approach to intervention targeting the youth environment within the Cherokee Nation. A computer-based intervention that incorporates developmentally appropriate gaming and video clips to prevent substance use (Project HAWK) also is being tested among AI youth (Raghupathy and Go Forth 2012). Researchers have not yet evaluated the efficacy of these new methods. Think Smart, another school-based program that develops both traditional and mainstream cultural competence among AI participants in the later elementary school grades, was associated with lower student inhalant abuse but showed null results for other substance use (Johnson et al. 2009).

Both Project HAWK and the Think Smart program were derived from the evidence-based State-wide Indian Drug Prevention Program that features skills training to increase bicultural competence and resilience among at-risk AI youth (Schinke et al. 2000). Use of innovative skills-training interventions is a fruitful area for improving prevention programs for other groups as well. For example, the REAL skills groups that focus on various refusal skills and a group-based social-norms approach have improved outcomes in the culturally based prevention program for Latino youth called Keepin’ It Real, especially when used with youth around the seventh grade (Marsiglia et al. 2012).

Beyond such adaptations of existing programs, other communities are experimenting with new methods developed in cooperation with minority groups themselves. For example, the Cherokee Talking Circle school-based intervention program, a uniquely Cherokee-centered strategy that includes the use of talking-circle groups as a culturally relevant approach to solving problems together, was associated with reduced substance use among AI youth. Those randomly assigned to the Cherokee Talking Circle intervention had significantly better outcomes with respect to total symptom severity, substance use, general life problems, and internal and external behavior at 3 months post-intervention than those assigned to a mainstream school-based substance abuse education program (Lowe et al. 2012).

Such CBPR among racial and ethnic minority populations has demonstrated the ethical and practical necessity of adaptive interventions that tend to evolve during the course of a research study. This can be done while preliminary outcomes are analyzed by researchers and community stakeholders and used to modify interventions (Henry et al. 2012). At the same time, some researchers have voiced concerns about overemphasizing the process of culturally adapting empirically validated mainstream interventions to the exclusion of other methods. One experienced AI research team (Whitbeck et al. 2012) urged a paradigm shift away from adapting Western best practices and toward development of novel evidence-based and culturally relevant interventions in partnership with Native communities. They suggested such a shift because interventions developed for Western populations sometimes do not align with Native worldviews and traditions. Moreover, many Native communities harbor a lingering deep distrust of Western-oriented practices because of historical abuses by researchers (Whitbeck et al. 2012).

### Advances in SBIRT and Motivational Interventions

Although novel, culturally based treatments ultimately may be considered ideal, mainstream SBIRT has been used successfully in racial and ethnic populations. One report (Madras et al. 2009) pooled data from multiple medical care settings (including emergency departments, primary care, and other institutions) for a study funded by SAMHSA to evaluate SBIRT, with the majority of the participating patients being people of color. The investigators found that, across the sites, patients experienced improved outcomes for substance-use and functional status 6 months post-intervention. Unfortunately, the types of brief interventions were not consistent across sites and there were no control groups, although all participating sites seemed to foster the spirit of motivational interviewing.

Brief motivational interventions with African Americans and Latinos in trauma centers also have been associated with reductions in alcohol use at 6 and 12 months post-intervention (Field et al. 2010). Ethnic matches between Latino clients and interventionists seemed to improve outcomes (Field and Caetano 2010), potentially supporting other research on the importance of the working alliance. Positive outcomes also did not depend on whether the subject subsequently attended treatment (Field et al. 2013).

Research from the National Institute on Drug Abuse (NIDA) Clinical Trials Network found that motivational enhancement therapy was particularly effective among African-American participants with higher readiness-to-change scores (Burlew et al. 2013). In a multisite randomized controlled trial, motivational enhancement therapy also was effective with and personally appealing to Spanish-speaking Latino adults who primarily misused alcohol, but less effective for those who used other drugs (Carroll et al. 2009). In another pilot study, culturally adapted motivational interviewing was well received by Latino immigrant participants (Lee et al. 2011).

## Other Advances in Treatment

Research studies have demonstrated empirical support for mindfulness-based relapse prevention as a substance-use intervention among women of color (Amaro et al. 2014; Witkiewitz et al. 2013; see sidebar “Religious Affiliation and Spiritual Practices: An Examination of the Role of Spirituality in Alcohol Use and Alcohol Use Disorder”). Although interest in using mindfulness as a substance-use intervention among racial and ethnic minorities has increased substantially, some researchers have raised questions about the cultural relevance of such interventions. For example, Hall and colleagues (2011) expressed concerns that mindfulness interventions may be highly Westernized. These strategies are not particularly helpful for certain racial and ethnic minority groups unless they are aligned with traditional cultural values and traditions.

Drink-refusal skills also have been identified as potentially helpful for African-American clients. In an examination of Project COMBINE data, African-American participants who completed drink-refusal skills training had significantly more positive treatment outcomes compared with those who did not complete the skills-training component. The positive outcomes were demonstrated up to 1 year post-intervention (Witkiewitz et al. 2011).

Communities also have collaborated with researchers using CBPR methods to create novel treatment interventions, just as they have done with prevention programs. One recent and promising example is the development of Drum-Assisted Recovery Therapy, which uses traditional Native American drumming and singing as well as talking circles to help AI/AN treatment clients with recovery from substance abuse (Dickerson et al. 2012). Researchers used qualitative methods and key community stakeholder involvement to develop and refine the culturally grounded therapy protocol that bears little resemblance to traditional treatment methods or mainstream therapies.

## Interventions for Sexual Minorities

Sexual minorities have been relatively overlooked in prevention and treatment intervention research, perhaps because of substance abuse stigma and homophobia. For sexual-minority clients of color, there also are the added dimensions of racial-and ethnic-based prejudice and bias. Sexual minorities experience elevated risk for substance abuse, but intervention research with this particular subpopulation is sorely lacking (Green and Feinstein 2012). However, researchers have found that in general, sexual-minority clients prefer to seek alternative rather than mainstream forms of treatment, especially if they do not closely identify with mainstream heterosexual beliefs (Dillworth et al. 2009).

Real Men Are Safe is a group-based program that emphasizes motivational enhancement, didactics, and skills training targeting high-risk sexual behavior among men in substance abuse treatment. It has been associated with modest improvements in safe-sex practices among sexual-minority men of color in substance abuse treatment. The program was culturally adapted by a qualitative examination of data collected from an expert panel of professionals who conducted research among ethnic sexual minorities that was then used to revise and enhance program content. Some evidence also suggests that the adapted Real Men Are Safe may have been more culturally relevant for African Americans and Latinos than for other groups (Calsyn et al. 2012, 2013). The results are promising and suggest that mainstream treatment can be culturally adapted for sexual-minority clients in ways that may reduce other risk behaviors.

## Advances in Pharmacologic Treatment

Beyond advances in psychotherapy, pharmacological approaches have been investigated in minority populations as well. In one randomized placebo-controlled trial with a rather high dropout rate, naltrexone use was associated with fewer alcohol-related consequences and greater percentage of days abstinent among AN clients in isolated rural areas of Alaska (O’Malley et al. 2008; see also Greenfield and Venner 2012).

However, two other studies found null results for naltrexone’s efficacy among African-American clients— one from Project COMBINE that examined alcohol-dependent participants (Ray and Oslin 2009) and another that investigated social drinkers under laboratory conditions (Plebani et al. 2011). Few pharmacotherapy studies have been conducted with minority population samples large enough to produce meaningful results. More investigation is needed to assess the efficacy of specific drugs, including naltrexone, among various subpopulations.

## Conclusions and Future Directions

Exciting new programs for prevention, brief opportunistic intervention, and treatment have been successfully developed and tested with racial, ethnic, and sexual minority populations—groups often at risk for substance abuse and with well-documented disparities. Recent interventions have combined computer- or Web-based technologies with culturally relevant adaptations, including a focus on the family as the unit of intervention, as well as culturally grounded and informed measurement (see Allen and Mohatt 2014). In addition, empirically supported skills-based approaches seem helpful for certain subpopulations, with the caveat that the interventions may require appropriate cultural alignment of the intervention with the beliefs and traditions of the group being targeted. Recent studies continue to demonstrate that when appropriate CBPR methods are used, evidence-based interventions can be used in culturally appropriate ways to benefit some racial, ethnic, and sexual minority populations.

However, given the vast heterogeneity of some minority groups (e.g., AI/AN) (Etz et al. 2012), some minority communities likely will reject existing interventions as culturally insensitive or not reflecting their beliefs and values (Whitbeck et al. 2012). In addition, some studies using culturally adapted interventions based on empirical evidence have found null or inconsistent outcomes (e.g., Carroll et al. 2009), suggesting that other approaches are needed. Thus, although such interventions can be helpful for some minority groups, a prudent strategy would involve simultaneously developing novel and culturally specific interventions using rigorous CBPR strategies for communities where other interventions may not work well (Etz et al. 2012; Whitbeck et al. 2012).

Intervening at the level of the treatment environment to improve outcomes for racial, ethnic, and sexual minority clients also is an exciting new development that holds particular promise for improving the working alliance, a consistent predictor of treatment outcomes independent of intervention modality. Above all, more can be done to improve the climate of prevention and treatment programs. Such efforts could reduce the likelihood of microaggressions and risk of stereotyping and stereotype threats that may negatively affect client outcomes following interventions.
